# Senior medical students as Trauma volunteers: is it worthwhile being on-call at a Level I Trauma Center?

**DOI:** 10.1186/1757-7241-21-S1-S25

**Published:** 2013-05-28

**Authors:** G Enomoto, P Abreu, A Nasr, F Tomasich, I Collaço, B Scheffer, L Bertholdi

**Affiliations:** 1 Médico pela UFPR - Turma 134. Brazil

## Introduction and Objectives

Medical students usually do supervised extra-curricular activities in different fields in Brazil. Doing these activities in the resuscitation room (RR) of a Level I Trauma Center is reserved for senior medical students (after 500 hours of extra-curricular activities done at the same hospital). The aim of this study was to assess the number of different procedures a senior medical student can develop in the RR over his extra-curricular activities in this environment.

## Methods

A retrospective observational descriptive study. Data were collected in the senior medical students’ registry book. After taking part in an activity, students registry the name of procedure, date, and the level of participation in a range of 4 possibilities. Statistical analysis was performed by the Google. docs online tool and percentage tests.

## Results

Over a period of 22 months, 1649 procedures performed by 79 senior medical students in 873 shifts (mean number of procedures per student = 20.87). In 64.22% of times, they did most of the procedure themselves. There were 4 Foley catheter placements, 6 CPRs, 1 gastric tube, and 12 central lines. Chest tube placement represented 9.21% of the procedures, being 76.31% of them properly performed by the supervised student. In 80% of times, the student stayed on call only doing no procedures at all. In 32.64% of shifts, the student could at least observe an exploratory laparotomy (scrubbing in and helping in 64.56% of them). In 51% of shifts, they were able to get in the Operating Room (scrubbed in and helping in 70.17% of times).

## Conclusions

Senior medical students on call play an important role in patient care and are exposed to a great variety of procedures. They can observe of even perform them supervised. We strongly recommend this kind of extra-curricular activity during the medical school, in order to students get manual dextricity and practical abilities.

